# A Dental Response to the COVID-19 Pandemic—Safer Aerosol-Free Emergent (SAFER) Dentistry

**DOI:** 10.3389/fmed.2020.00520

**Published:** 2020-08-12

**Authors:** Habib Benzian, Richard Niederman

**Affiliations:** WHO Collaborating Center Quality Improvement & Evidence-Based Dentistry, Department Epidemiology & Health Promotion, College of Dentistry, New York University, New York, NY, United States

**Keywords:** COVID-19, infection control, pandemic preparedness and response, infectious dental aerosols, airborne transmission of diseases, dental care, emergency dental service

## Abstract

Dental services are significantly impacted by the COVID-19 pandemic. Almost all dental procedures carry a high infection risk for providers and patients due to the spread of aerosols. As a consequence, public health agencies and professional associations have issued guidelines for enhanced infection control and personal protection equipment and have also limited care to urgent or emergency services. However, there is no dental service concept for pandemic disaster preparedness or response that might be applied. Moreover, pathways to dental care provision in a post-pandemic future with persisting risks are needed. We propose *Safer Aerosol-Free Emergent Dentistry* (SAFER Dentistry) as one approach to dental services during and emerging from the pandemic. The concept's starting point is the identification of the most common patient needs. The next step is to replace common treatments addressing the most frequent needs with alternative interventions involving a lower infection risk because they do not generate aerosols. SAFER Dentistry is innovative, avoids risk, and responds to the requirements of a pandemic and post-pandemic emergency where the risk of airborne disease transmission remains high. SAFER Dentistry thereby ensures continuity of dental services while protecting providers and patients from infectious pathogens. Moreover, SAFER Dentistry allows dental service providers to remain operational and generate income even under pandemic conditions. Potential implementation and policy options for SAFER Dentistry include universal availability without co-payments by patients and a uniform bundled payment scheme for providers to simplify budgeting, reimbursement, and administration during a pandemic. Adaptations and adjustments of the concept are possible and encouraged as long as the principle of avoiding aerosol-generating procedures is maintained.

## SARS-CoV-2 Disrupts Health Care and Dentistry Worldwide

*In a crisis, be aware of the danger, but recognize the opportunity*. John F. Kennedy.

Healthcare services are adapting to the COVID-19 pandemic, yet oral health care and dentistry are particularly affected due to the proximity to the patient and the generation of aerosols through common treatment procedures. The SARS-CoV-2 virus seriously disrupts routine dental procedures around the world. The reports, analyses, and recommendations emerging alongside first-hand experience from dental settings in China are alarming (1, 2). The risks of infection for oral health personnel as well as cross-infection between patients and anyone in a dental care setting are high.

The toll of the COVID-19 pandemic on health systems and availability of dental care has been unprecedented. At the height of the pandemic in the U.S., about 80% of practices offered only limited emergency services, and 17% did not see patients at all (3). This impacts heavily on service availability and patient access to essential services. At the same time, such service limitations result in significant economic losses for the entire dental sector, leading to layoffs of dental teams and growing unemployment. In addition, the prospect of re-starting services remains bleak and uncertain and will be so for months to come (4). Little is known so far about the impact on dental services in low- and middle-income countries, though recommendations for service limitations to emergency care and increased precautions were issued in many countries (5).

## The Infection Risk From Aerosols in Dentistry

Dental teams are generally used to high standards of infection control and personal protection measures owing to the fact that dental personnel are among the most at risk for any kind of infection transmitted via contaminated aerosols and saliva, bodily fluids, blood, or tissue particles (6, 7).

Current evidence suggests three main pathways for virus transmission in dental settings: (1) direct transmission through inhalation of cough, sneeze, or droplets containing the virus; (2) transmission via eye, nasal, or oral mucous membranes; and (3) contact transmission through contaminated surfaces (2). All these transmission pathways are facilitated and possibly amplified by aerosols that are generated by most dental procedures (7, 8).

In reaction to the COVID-19 pandemic, international and U.S. federal public health agencies, as well as dental professional associations, have issued specific guidance for the control of SARS-CoV-2 in dental practice (5, 9–13).

These recommendations focus on three main areas where adaptation to the pandemic context is required to break potential transmission chains: patient management and teledentistry to prevent sick or possibly infected patients from coming to the practice, enhanced infection-control measures that include strict protocols for personal protective equipment (PPE), and limitation of dental care to urgent and emergency procedures. Some authorities demand that for patients with COVID-19 symptoms, emergency oral health care should be performed in a negative-pressure operatory with maximum PPE to reduce infectious health hazards (14, 15). Countries like the UK have therefore established special dental care centers to ensure appropriate protection (16).

In the U.S., the Occupational Safety and Health Administration (OSHA) considers work environments where aerosols may occur to be of high or very high infection risk for COVID-19 (14). In a specific update for dentistry, the OSHA requires telephone triage, office engineering controls that include air circulation and patient isolation, universal precautions for airborne pathogens, the use of PPE appropriate for the pandemic, limiting care to urgent and emergency procedures that do not generate aerosols, and environmental cleaning post-care. The recommendation for airborne infection isolation rooms (AIIR) with negative pressure is in line with the U.S. Centers for Disease Control and Prevention's (CDC) existing guidance (17).

Such measures beyond the standard dental infection-control procedures are challenging due to limited or costly supplies of PPE, and they would require significant infrastructure investments. Many of the requirements are unrealistic to achieve even in sophisticated university dental college settings, at least in the short term. For many dental care contexts, such as mobile dental services for schools, remote communities, nursing homes, prisons, homeless shelters, or refugee camps, as well as dental services in low-resource settings where the shortage of supplies is a constant challenge, such enhanced protective measures are near impossible.

The risks from infectious aerosols are central to all recommended alterations of current dental practice, yet uncertainties and open questions related to transmission details remain and oblige dental practitioners to assume they are operating under the highest possible infection risk and to act accordingly with appropriate precautions (18).

Dentistry as we know it is seriously disrupted and may not be able to return to the clinical routines of a pre-COVID-19 time. At this point of the pandemic, dentistry needs a concept for continued dental services that avoids procedures generating infectious aerosols while being able to address the most frequent patient oral health needs.

## Safer Aerosol-free Emergent (SAFER) Dentistry

With aerosol-generating procedures being at the core of the current challenge for dental services, interventions that avoid aerosol generation should be the interventions of choice. Such procedures exist and may replace possibly hazardous “standard” therapies in an emergency context with airborne pathogens such as SARS-CoV-2. We propose the concept of *Safer Aerosol-Free Emergent Dentistry* (SAFER Dentistry). SAFER Dentistry builds on a prioritization of the most common patient needs, and systematically selects bundles of effective, evidence-based, and value-based care that do not require aerosol-generating procedures.

Focusing on emergency and urgent dental services, SAFER Dentistry addresses common care scenarios with a set of bundled interventions. Table 1 details the treatment options with significantly lower risk of generating aerosols, including scientific references for the respective non-aerosol options. They comprise the following.

1. Examination/diagnosis via in-person teledentistry: when performed in person, this includes antiseptic mouthrinse and visual and/or tactile inspection without intraoral radiography for diagnosis.
2. Acute pain, swelling, or infection: depending on the diagnosis, pulp devitalization/temporary filling (pulpitis), antibiotic therapy (acute inflammation), and/or local anesthesia and tooth extraction.
3. Toothache due to caries without pulpal involvement: silver-diamine-fluoride application (SDF), glass-ionomer sealants/Atraumatic Restorative Treatment (ART), fluoride varnish/gel, and/or toothbrushing with high fluoride-containing toothpaste (HFT, 5,000 ppm fluoride).
4. Acute periodontitis: hand scaling and metronidazole/amoxicillin combination for 1 week.
5. Denture repair/reline, lost crown or orthodontic bracket, or orthodontic wire: denture repair with soft re-line, crown and bracket re-cementation, and wire adjustment, repair, or removal as well as removal of stiches from previous surgery.

**Table 1:** SAFER Dentistry Packages and intervention options.

	**Package**	**Intervention options without aerosol risk**	**Conventional options with aerosol risk**	**References**
1	Examination	• Teledentistry–remote triage, examination and counseling • Pre-examination antiseptic mouthrinse • Visual examination • Examination with instruments ° Probing, percussion test ° Pulp vitality testing (ice-pellet/heated gutta-percha/electric testing) • Extraoral X-ray if available and required (OPG)	• Intraoral x-ray (risk of avulsion & coughing) • Temperature test with cold air blow (saliva splatter) • Tactile examination/palpation	(19) (2) (20) (21) (1) (22)
2	Pain: Swelling & infection	• Local anesthesia • Incision of abscess & drainage • Or/and antibiotic therapy • Or tooth extraction (avoiding surgical separation or drilling)		(23) (1)
	Pain: Toothache & pulpitis	• Local anesthesia • Trepanation/opening of pulp chamber with hand instrument (excavator), extirpation & disinfection of root canal, temporary filling • Or tooth extraction (avoiding surgical separation or drilling)	• Trepanation with drill & spray • Machine preparation and cleaning of root canals	(22) (23) (24) (1)
3	Pain: Toothache & caries Caries prevention	• Silver diamine fluoride (SDF) • Glass ionomer sealants • Atraumatic Restorative Treatment (ART) with glass ionomer • Fluoride varnish • Fluoride gel/5,000 ppm F toothpaste	• Caries excavation & traditional restorative care (drilling & filling)	(25) (26) (27) (28) (29)
4	Acute periodontitis/pericoronitis	• Cleaning and scaling with hand instruments • Antibiotic therapy (if indicated) • Antiseptic mouthrinse/gel (i.e., CHX)	• Ultrasonic scaling and machine polishing	(22) (1) (30)
5	Broken denture Orthodontic emergency & post-surgery care	• Direct reline/rebase • Removal/adjustment of broken orthodontic wire causing serious irritation • Removal of stitches from previous surgery	• Indirect repair with impression/laboratory technician (risk of avulsion & coughing)	(31) (22)

The interventions of SAFER Dentistry are effective and realistic, even for resource-poor settings. Individually, they have been used for decades and have been promoted widely (30). The systematic bundling and prioritization, however, is new and innovative and responds to a pandemic and post-pandemic context where the risk of disease transmission remains high or might be intermittently increasing or decreasing. This approach ensures that dental services can continue during a pandemic by providing oral health care for the most frequent patient needs, while protecting providers and patients from pathogens. Dental teams will require little to no additional training to perform the interventions of SAFER Dentistry, since none of the procedures are new or unknown.

There is no clinical dental care situation without any infectious risk. The alternative interventions suggested are not completely risk-free, but carry a significantly lower and more manageable risk. We therefore use the acronym SAFER Dentistry in analogy to the HIV pandemic's response concept of *Safer Sex*, which also significantly reduces the risk of transmission of HIV and other sexually transmitted diseases (32).

## Healthy System Options for Implementing SAFER Dentistry

Pandemics are a constant challenge to public health and reveal with relentless clarity the shortcomings of health systems in terms of capacity, coverage, quality, and financing. The same applies to inequalities and differential impact of the pandemic on different population groups. Oral health status and access to dental care has long been recognized as a prime example for such challenges (33). With millions of people unemployed due to the COVID-19 pandemic, many are losing their health insurance benefits at a time when most needed. There is growing recognition that basic health (and oral health) services are a public good that should be universally available for everyone, irrespective of their employment status (34). SAFER Dentistry, covering the most frequent oral health needs, is one starting point for a basic oral health benefit package. Further adaptations and evolving implementation may also include aerosol-free cavity prevention (dental sealants and the like) and other preventive measures to reduce the need for dental care as pandemics continue to emerge. In order to ensure maximum population coverage, we propose that SAFER Dentistry be universally available with no co-payments. Initial economic modeling for children shows that SAFER Dentistry is cost-saving and cost-effective compared to conventional aerosol-generating interventions (35).

Providers could be reimbursed through a single, uniform payment for any combination of examination and additional procedure, thereby simplifying documentation, billing, and reimbursement, which is of particular importance in an emergency context. Such an approach would work for health systems relying on a fee-for-service approach as well as for capitation-based systems. However, the details of the required changes in guidelines, service directives or other adaptations need to be determined nationally/locally depending on existing conditions, resources, local guidelines, and political support. Aspects of teledentistry should be included in the benefit package since they will become a more frequent practice and specific reimbursement positions are often not yet in place (19).

As the pandemic and related limitations of clinical practice endure, more and more patients will have to use emergency hospital services for relief of their dental problems. In the US, even without a pandemic, every 15 s a patient visits a hospital emergency service for dental care due to millions lacking dental insurance coverage (36). Universally available SAFER Dentistry would reduce such hospital visits for common dental ailments and unburden hospital personnel, infrastructure, and resources; it would instead offer an opportunity for dental service providers to remain operational and generate income. At the same time, SAFER Dentistry allows providers to offer a safe and hygienic service environment as a key component to regaining patient trust in the period of pandemic recovery.

## Conclusions

The early experiences in dentistry from China during this COVID-19 pandemic are instructive and telling: They implemented rapid and bold actions to contain the pandemic, including limited emergency dental services in a tertiary care center with maximum precautions (1). The UK and other countries also established specialized emergency care centers for dentistry (37, 38). Yet, the level of infrastructure and service provisions possible in such centers are not realistic for general dental practices or oral health training programs in the U.S. or globally.

SAFER Dentistry, together with general measures to mitigate risk in dental settings, is an adaptation to a pandemic emergency and a pandemic recovery process by avoiding hazardous infectious aerosols. It is also a first step toward oral health care that does not require complex technology, as envisaged in the landmark Lancet Series on Oral Health (39). If continued and institutionalized as a universally available benefit and coverage feature, substantial gains in oral health status and significant reductions in oral health care expenditure could be achieved in the long run.

The dangers of the crisis are clear. Continuation of dentistry as usual during the COVID-19 pandemic will result in incalculable risks for patients and providers. For U.S. governmental agencies and professional organizations, oral healthcare training institutions, clinicians, and patients not willing to accept a complete shutdown of oral healthcare, including deterioration of health and well-being, there is no alternative to SAFER Dentistry.

## Data Availability Statement

The original contributions presented in the study are included in the article/supplementary material, further inquiries can be directed to the corresponding author/s.

## Author Contributions

All authors listed have made a substantial, direct and intellectual contribution to the work, and approved it for publication.

## Conflict of Interest

The authors declare that the research was conducted in the absence of any commercial or financial relationships that could be construed as a potential conflict of interest.
